# The effects of video games on cognitive function in older adults with mild cognitive impairment: a meta-analysis

**DOI:** 10.3389/fnagi.2025.1756970

**Published:** 2026-01-23

**Authors:** Bo Liu, Xiaomei Li, Hejia Cai

**Affiliations:** 1 Outdoor Sports Academy, Guilin Tourism University, Guilin, China; 2 Guilin Municipal Hospital of Traditional Chinese Medicine, Guilin, China

**Keywords:** cognitive function, exergames, meta-analysis, mild cognitive impairment, video games

## Abstract

**Objective:**

This study aimed to evaluate the efficacy of video-game–based interventions in enhancing global cognitive function and executive functioning among individuals with mild cognitive impairment (MCI).

**Methods:**

A systematic search was conducted across PubMed, Web of Science, The Cochrane Library, Embase, CINAHL, and Medline, covering all publications available up to October 12, 2025. Meta-analyses were performed using Review Manager 5.3 and Stata 17.

**Results:**

Five randomized controlled trials were ultimately included, comprising approximately 215 participants. Meta-analytic findings revealed that, compared with control conditions, video-game interventions significantly improved global cognition, reflected by increases in Montreal Cognitive Assessment (MoCA) scores (MD = 2.58, 95% CI: 1.27–3.90, *P* < 0.0001) and Mini-Mental State Examination (MMSE) scores (MD = 1.80, 95% CI: 0.79–2.80, *P* = 0.0005). Executive function and attentional performance also showed marked enhancement, evidenced by substantial reductions in completion time on the Trail Making Test A (TMT-A) (SMD = −1.38, 95% CI: −1.73 to −1.04, *P* < 0.00001) and Trail Making Test B (TMT-B) (SMD = −3.50, 95% CI: −6.03 to −0.98, *P* = 0.007).

**Conclusion:**

Video-game–based interventions appear to meaningfully enhance both global cognitive function and executive functioning in individuals with MCI. However, the limited number of included studies underscores the need for future research to establish standardized intervention protocols and incorporate extended longitudinal follow-up.

**Systematic review registration:**

https://www.crd.york.ac.uk/PROSPERO/view/CRD42024539189, identifier: CRD42024539189.

## 1 Introduction

Dementia, a prevalent chronic neurodegenerative disorder in older adults, has emerged as the fourth leading cause of mortality worldwide, following malignancies, heart disease, and stroke. By 2050, the global population affected by dementia is projected to reach 150 million, approximately tripling the current burden (Nichols et al., 2022). Beyond imposing profound economic strain on patients, caregivers, and society at large, dementia detrimentally affects psychological wellbeing as well as social and physiological functioning; nevertheless, no curative treatment is available to date (Bai et al., 2022). Given the irreversible trajectory of dementia and the limitations of existing therapies, early intervention has become a central focus of both prevention and management strategies.

Mild cognitive impairment (MCI), an intermediate stage between normal aging and dementia, is initially characterized by subtle memory decline and gradually progresses to severe cognitive impairment, ultimately advancing to dementia (Petersen et al., 2014). Epidemiological evidence indicates that the global prevalence of MCI ranges from 15.56% to 19.7% (Song et al., 2023), with an annual conversion rate to dementia of 10%−15%, far exceeding the 1%−2% incidence observed in the general population (Chen et al., 2022). As a critical window for dementia prevention, the MCI stage is particularly responsive to targeted therapeutic strategies that may delay or avert disease progression. In the absence of pharmacological agents capable of halting or reversing the disease course, non-pharmacological interventions—valued for their safety, strong adherence, and cost-effectiveness—have garnered increasing attention (Petersen et al., 2018). Among these approaches, cognitive training and physical exercise represent two primary modalities. Cognitive training aims to strengthen specific cognitive skills through repetitive, progressively challenging mental tasks, whereas physical exercise has been shown to enhance cardiovascular function, increase cerebral perfusion, and stimulate the release of brain-derived neurotrophic factor (BDNF), thereby promoting neurogenesis and synaptic plasticity (Bherer, 2015). Mounting evidence suggests that cognitive or physical interventions alone may yield limited benefit, whereas dual-task or combined training paradigms—integrating cognitive stimulation with motor activity—can elicit superior neuroprotective and cognitive-enhancing effects (Shatil, 2013). Such combined models are believed to act synergistically by concurrently activating distinct neural circuits, thereby reinforcing neuroplastic processes and more effectively mitigating deficits in executive function and processing speed characteristic of individuals with MCI.

Video games encompass all games played on digital platforms, including arcade systems, personal computers, dedicated gaming consoles (e.g., Nintendo GameCube, Sony PlayStation, Microsoft Xbox), and handheld devices (e.g., Nintendo Game Boy, Sony PSP) (Baranowski et al., 2008). Video games are a dominant subclass within this broader category, specifically referring to electronic games that rely on video displays as their primary visual output, generating rasterized signals to deliver dynamic imagery through screens such as CRT, LCD, or OLED monitors. Most contemporary gaming forms fall squarely within this definition. As an emerging technological platform well-suited to dual-task interventions, video games allow users to interact with immersive virtual environments through gross motor movements using systems such as Xbox 360 Kinect, Nintendo Wii, or modern virtual reality (VR) devices. This approach inherently integrates physical activity with continuous, cognitively demanding tasks—such as visual search, decision-making, and rapid response—thereby fulfilling the requirements of combined physical–cognitive stimulation.

Although entertainment rather than training is the primary motivation for playing video games, their engaging nature has increasingly supported their use in cognitive rehabilitation. Numerous studies have demonstrated that video games enhance cognitive control, working memory, and episodic memory in older adults (Anguera et al., 2013; Toril et al., 2016), and may also alleviate depressive symptoms while improving subjective wellbeing (Bergmann et al., 2023). Among individuals with MCI, video-game interventions have been shown to bolster global cognition and prefrontal executive functioning (Sato et al., 2023; Choi et al., 2025). However, existing findings remain inconclusive; one meta-analysis reported that video-game training failed to produce measurable cognitive improvements (Sala et al., 2018).

To provide a more comprehensive assessment of the cognitive effects of video-game interventions in individuals with MCI, the present study conducted a meta-analysis synthesizing available evidence. By objectively evaluating the impact of video games on cognitive outcomes, this analysis aims to furnish more substantive clinical guidance regarding their utility in cognitive rehabilitation for MCI.

## 2 Methods

This systematic review and meta-analysis was reported according to the Preferred Reporting Items for Systematic Reviews and Meta-Analyses (PRISMA) guidelines (Page et al., 2021). The review panel conducted the systematic review and meta-analysis in accordance with the PRISMA checklist This protocol was registered in the Prospective Register of Systematic Reviews (http://www.crd.york.ac.uk/PROSPERO, ID: CRD42024539189) before the review was conducted.

### 2.1 Search strategy

All search procedures were conducted in accordance with the PRISMA 2020 guidelines (Sarkis-Onofre et al., 2021) and completed prior to October 12, 2025. A systematic search was performed using MeSH-based keywords including “mild cognitive impairment,” “video game,” and “cognitive function” across PubMed, Web of Science, The Cochrane Library, Embase, CINAHL, and Medline. In addition, Google Scholar and the reference lists of all retrieved articles were manually screened to identify any potentially relevant studies. All collected citations were imported into EndNote X9 for further evaluation. A more detailed search strategy is in Supplementary material.

### 2.2 Inclusion and exclusion criteria

Titles and abstracts of retrieved publications were independently screened by— two researchers, followed by full-text assessment to determine eligibility. All included studies met the PICOS-based criteria as follows:

Participants (P): older adults aged ≥ 50 years diagnosed with mild cognitive impairment, irrespective of sex;
Intervention (I): video-game–based interventions;
Comparison (C): no intervention or health education/usual care alone;
Outcomes (O): global cognitive function and/or memory, executive function, attention;
Study design (S): randomized controlled trials (RCTs).
Language: publications in English (full-text available in either language).

Exclusion criteria were:

① Studies involving participants diagnosed with Alzheimer's disease, other types of dementia, or cognitive impairment secondary to other neurological or psychiatric disorders (e.g., stroke, depression, Parkinson's disease);
② Studies reporting outcomes unrelated to cognitive performance (e.g., non-cognitive endpoints);
③ Studies with insufficient extractable data or unavailable full texts.

### 2.3 Study selection and data extraction

Two researchers independently performed study selection according to the predefined criteria, with discrepancies resolved through consultation with a third reviewer. Extracted information included: (1) basic study characteristics (authors, publication year); (2) intervention details (type, duration, frequency, and session length); (3) participant characteristics (age, sex); and (4) outcome measures.

### 2.4 Quality assessment

The methodological quality of included RCTs was evaluated using the Cochrane risk-of-bias tool, encompassing seven domains: random sequence generation, allocation concealment, blinding of participants and personnel, blinding of outcome assessment, completeness of outcome data, selective reporting, and other potential sources of bias. Each domain was rated as “low risk,” “high risk,” or “unclear,” and a corresponding risk-of-bias graph was generated.

### 2.5 Statistical analysis

All outcome measures were treated as continuous variables, and intervention effects were expressed as standardized mean differences with 95% confidence intervals. Effect models were selected based on heterogeneity: fixed-effects models were applied when *I*^2^ ≤ 50%, whereas random-effects models were used when *I*^2^ > 50%. Sensitivity analyses were performed by sequentially removing individual studies to evaluate their influence on overall results. Subgroup analyses were conducted to explore potential sources of heterogeneity. Meta-analyses were performed using Review Manager 5.3 and Stata 17.0.

## 3 Results

### 3.1 Study selection

**Figure 1:**
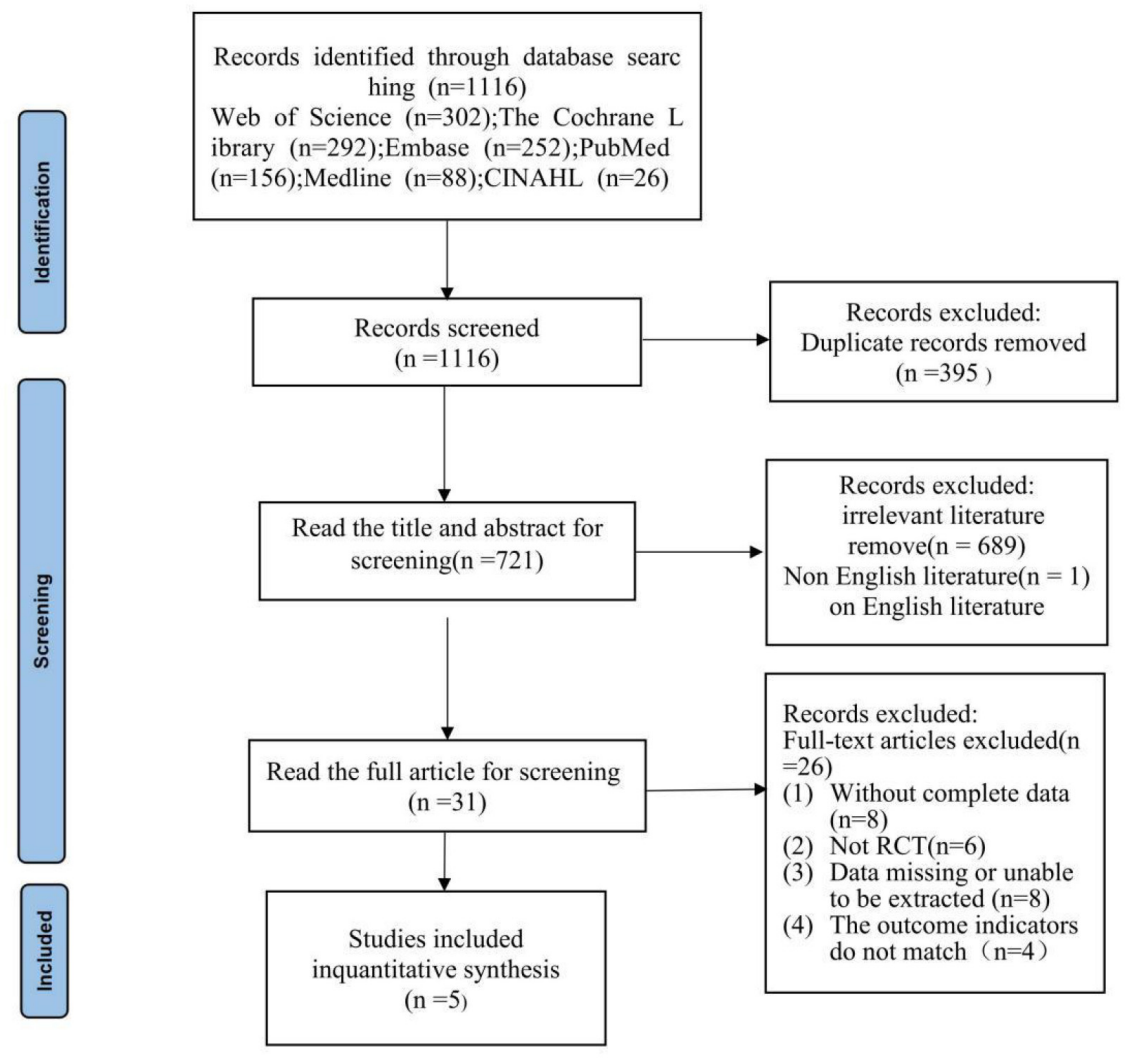
Study selection represented by PRISMA flowchart.

The initial search yielded 1,116 records across domestic and international databases. After deduplication using EndNote and manual procedures, 721 records remained. Two reviewers independently screened titles and abstracts, excluding 690 records that did not meet the inclusion criteria. Thirty-one articles were assessed in full, and 5 studies were ultimately included, encompassing 215 participants. The study selection process is shown in Figure 1.

### 3.2 Characteristics of the included studies

Across the five randomized controlled trials, four employed exergaming (motion-sensing video games) as the experimental intervention, and one used tablet-based (iPad) games. Control conditions consisted of either no intervention or exercise alone. Intervention durations ranged from 4 to 12 weeks; the remaining three trials implemented 6-weeks programs. Training frequency was typically 3 or 5 sessions per week, with individual sessions lasting 30–60 min. Regarding outcomes, the Montreal Cognitive Assessment (MoCA), Trail Making Test-Part A (TMT-A), and Trail Making Test-Part B (TMT-B) were the most frequently used cognitive measures, collectively applied in four trials; three trials also administered the Mini-Mental State Examination (MMSE). See Table 1.

**Table 1:** The details of research characteristics.

**Included studies**	**Sample size (T/C)**	**Mean age, years (T/C)**	**Intervention (T)**	**Intervention (C)**	**Frequency/ week**	**Exercise time/ day**	**Exercise cycle**	**Outcome measure**
Aruba Saeed, 2024	65/22	69.0 ± 6.8/72.32 ± 7.7	Wobble-board exergame balance training	Wii Fit balance training	3 times/w	40 min	8 w	MoCA, TMT-A, TMT-B
George Savulich 2017	21/21	75.2 ± 7.4/76.9 ± 8.3	iPad memory-game cognitive training	Routine outpatient follow-up	8 times/4 w	60 min	4 w	MMSE
Hafsah Arshad 2023	26/25	62.85 ± 5.56/63.24 ± 5.12	Xbox 360 Kinect motion-based cognitive games	Upper-/lower-limb stretching and theraband resistance training	5 times/ w	30 min	6 w	MMSE, MoCA, TMT-A, TMT-B
Chien-Liang Liu 2022	16/17	74.6 ± 6.1/73.4 ± 6.5	Motion-sensing tai chi	No intervention; usual activities maintained	3 times/ w	50 min	12 w	MoCA, TMT-A, TMT-B
Imran Amjad 2019	22/22	—/—	Xbox 360 Kinect motion-based cognitive games	Range-of-motion and stretching training	5 times/ w	25–30 min	6 w	MMSE, MoCA, TMT-A, TMT-B

T: Treatment group group,; C: Control group,; w: week.

### 3.3 Risk-of-bias assessment

**Figure 2:**
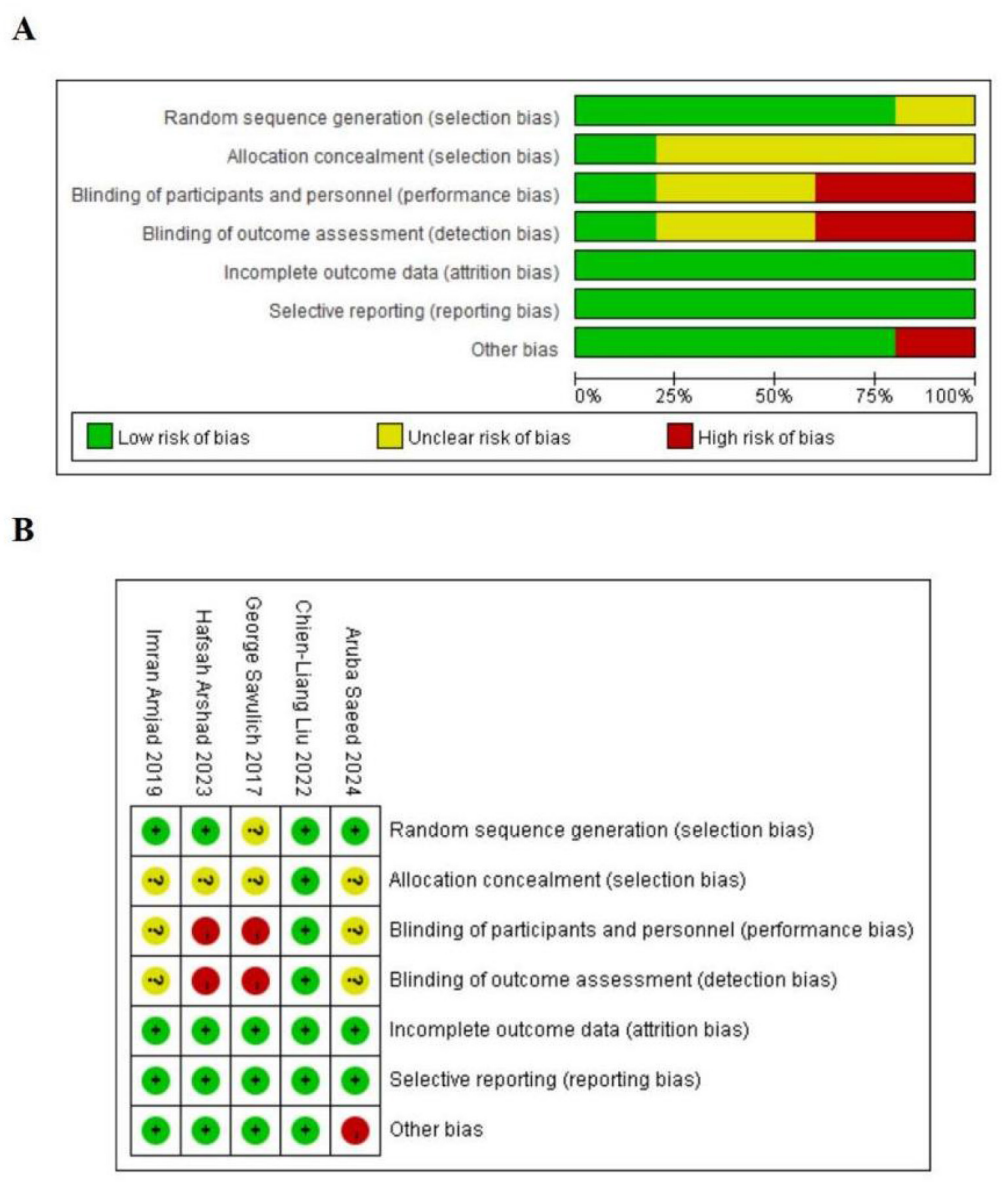
**(A)** Risk of bias summary; **(B)** risk of bias assessments.

Risk of bias was evaluated using the Cochrane Risk of Bias 2 (RoB 2) tool, with findings summarized in Figure 2. Overall methodological quality was relatively high. Three trials explicitly reported random sequence generation, and one described allocation concealment. Owing to the nature of the intervention, blinding of participants and personnel was impracticable, conferring an elevated risk of performance bias (bias due to deviations from intended interventions). One study was judged at high risk for other bias because of a markedly imbalanced sex distribution, with a predominance of male participants. The remaining four trials were considered at low risk of other bias.

**Figure 3:**
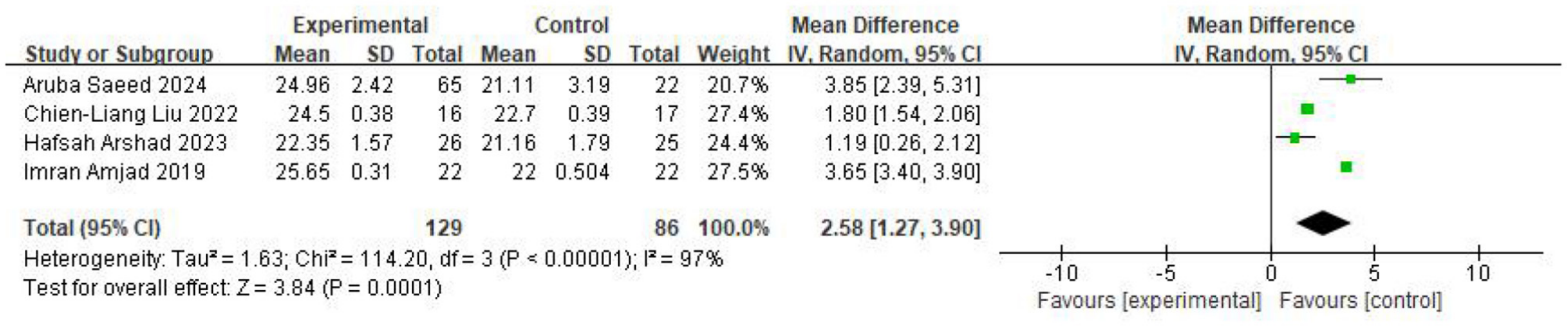
Forest plot of the meta-analysis evaluating the effect of video game interventions on MoCA scores.

Across four studies assessing the MoCA scale, including 129 participants in the intervention group and 86 in the control group, the pooled analysis using a random-effects model demonstrated a significantly higher MoCA score in the intervention group (MD = 2.58, 95% CI 1.27–3.90, *P* < 0.0001; *I*^2^ = 97%). The results were displayed in Figure 3.

**Figure 4:**
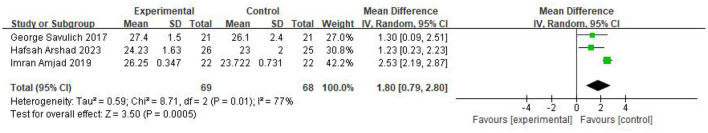
Forest plot of the meta-analysis evaluating the effect of video game interventions on MMSE scores.

Three studies evaluated the MMSE, comprising 69 participants in the intervention group and 68 in the control group. The results, derived from a random-effects model, revealed that the intervention group exhibited significantly higher MMSE scores compared with the control group (MD=1.80, 95% CI 0.79–2.80, *P* = 0.0005; *I*^2^ = 77%). The results were displayed in Figure 4.

**Figure 5:**
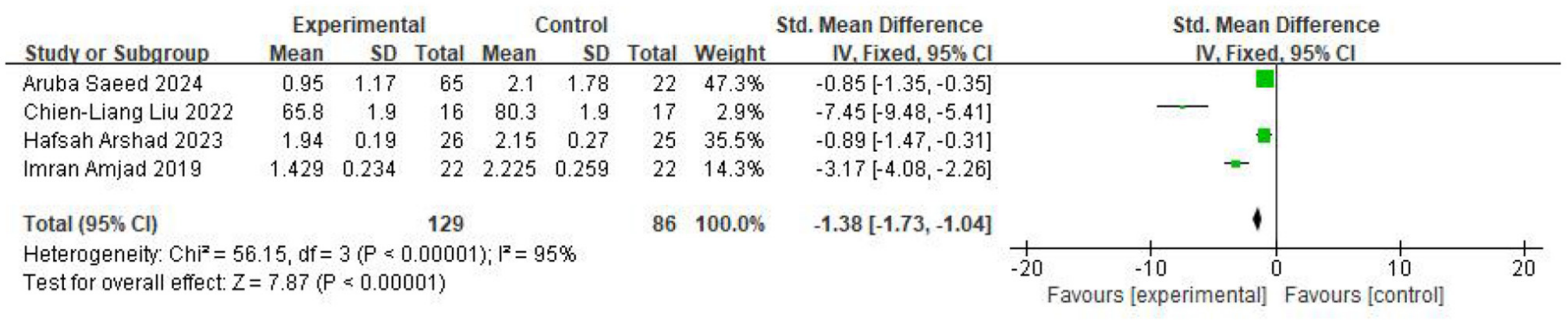
Forest plot of the meta-analysis evaluating the effect of video game interventions on TMT-A scores.

Four studies reported outcomes for the TMT-A, with 129 participants in the intervention group and 86 in the control group. The findings from the random-effects model indicated that the intervention group had markedly lower TMT-A scores (indicating better performance) than the control group (SMD = −1.38, 95% CI −1.73 to −1.04, *P* < 0.00001; *I*^2^ = 95%). The results were displayed in Figure 5.

**Figure 6:**
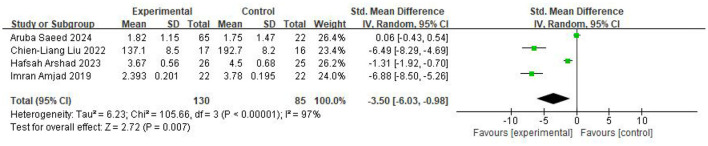
Forest plot of the meta-analysis evaluating the effect of video game interventions on TMT-B scores.

Four studies assessed the TMT-B, involving 130 participants in the intervention group and 85 in the control group. The pooled results (random-effects model) showed that the intervention group demonstrated substantially lower TMT-B scores compared with the control group (SMD = −3.50, 95% CI −6.03 to −0.98, *P* = 0.007; *I*^2^ = 97%), reflecting significant improvement in executive function. The results were displayed in Figure 6.

### 3.4 Subgroup analysis

Subgroup analyses were conducted to explore heterogeneity based on control condition (active vs. passive), intervention duration (≤6 weeks vs. >6 weeks), and session frequency.

#### 3.4.1 MoCA

Significant subgroup differences were found for control type (*P* < 0.001) and frequency (*P* < 0.001). Studies employing active controls (e.g., stretching, balance) showed significantly larger effect sizes (MD = 3.50) compared to passive controls (MD = 1.80). Similarly, high-frequency interventions (>3 sessions/week) yielded greater cognitive benefits (MD = 3.49) compared to lower frequency (MD = 1.86).

#### 3.4.2 MMSE

Although active controls (MD = 2.40) and motion-based exergames appeared to produce larger improvements than passive/static controls (MD = 1.30), the difference was not statistically significant (*P* = 0.086), likely due to the small number of studies.

#### 3.4.3 Executive function (TMT-A&B)

For TMT-B, shorter duration and higher frequency interventions were associated with significantly larger effect sizes (*P* < 0.001). Interestingly, for TMT-A, the passive control subgroup showed an unusually large effect size, driven by one study (Liu et al., 2022), which contributed to significant heterogeneity (*P* < 0.001).

### 3.5 Sensitivity analysis

Because considerable heterogeneity was detected in the results of the MMSE, MoCA, TMT-A, and TMT-B assessments, a leave-one-out sensitivity analysis was performed. This approach aimed to evaluate the influence of each individual study on the pooled effect estimates, to better understand the sources of heterogeneity, and to identify potential outliers. The MMSE outcomes demonstrated pronounced heterogeneity across studies (*I*^2^ = 78%). After sequentially excluding the three MMSE studies, the analysis indicated that the study by Amjad et al. (2019) contributed most to the heterogeneity and was therefore removed from the meta-analysis. Following its exclusion, heterogeneity was eliminated (*I*^2^ = 0%), and the pooled effect remained statistically significant (95% CI 0.49–2.03).

In contrast, for the MoCA, TMT-A, and TMT-B outcomes, heterogeneity persisted despite stepwise exclusion of individual studies, indicating that the findings of the present meta-analysis are robust.

### 3.6 Publication bias

As fewer than 10 studies were included, the possibility of publication bias cannot be ruled out.

## 4 Discussion

This study demonstrates that video games can enhance global cognitive functioning in individuals with MCI, underscoring their therapeutic potential for MCI and their preventive value in delaying dementia. These findings align with recent evidence across multiple studies (Ballesteros et al., 2014; Yang and Kwak, 2017; Yen and Chiu, 2021; Nath et al., 2023; Sato et al., 2023; Choi et al., 2025), indicating that video games are not merely a form of entertainment but a clinically promising tool for cognitive enhancement. The mechanisms underlying such improvements are multifaceted, potentially involving the induction of structural brain plasticity, increased neural network efficiency, and modulation of dopaminergic pathways.

The significant improvement in global cognition (MoCA and MMSE) observed in our study aligns with the hypothesis that video-game interventions may drive neuroplastic changes. While our meta-analysis focused on behavioral outcomes, previous literature suggests that navigating complex 3D environments—similar to those in the included exergaming studies—can stimulate hippocampal function and promote structural integrity in regions critical for memory and executive control (West et al., 2017; Clemenson et al., 2020; Clemenson and Stark, 2015). These underlying neural mechanisms may explain the observable gains in cognitive scores reported here.

Global cognition represents an integrated constellation of cognitive domains, including attention, memory, and executive function. Video games—particularly the motion-based and action-oriented games frequently represented in our included studies—constitute an intensive form of “multidomain cognitive training.” Gameplay requires sustained attention, rapid visual search, engagement of working memory, and dynamic motor planning, whether through controller manipulation or full-body movement. A seminal Nature study by Anguera et al. (2013) demonstrated that such multitasking training enhances frontal theta-band activity, improving not only task-specific performance but also enabling far transfer to untrained domains such as working memory and sustained attention, thereby strengthening the core executive control network that underlies higher-order cognition.

Most studies included in our analysis employed motion-based games, which integrate physical activity with cognitive challenge to create a dual-task training environment. Aerobic exercise alone can increase cerebral blood flow, improve cerebrovascular health, and upregulate neurotrophic and growth factors such as BDNF and IGF-1, providing a metabolic milieu conducive to neurogenesis (A review of physical cognitive interventions in aging, 2014). When layered with cognitive demands such as visual search, inhibitory control, and response planning, motion-based games harness this neuroplastic potential to guide synaptic remodeling within targeted neural networks. The “cybercycling” paradigm investigated by Anderson-Hanley et al. exemplifies this synergy: compared with standard cycling, cycling augmented with virtual-reality cognitive tasks significantly elevated plasma BDNF levels and yielded greater improvements in executive function among MCI patients (Anderson-Hanley et al., 2012) In line with Bamidis et al.'s (2014) “physical–cognitive synergy hypothesis,” aerobic exercise supplies the neurobiological resources—via increased cerebral perfusion and upregulation of IGF-1 and VEGF—while concurrent cognitive gaming shapes the direction of plastic change through guided plasticity (A review of physical cognitive interventions in aging, 2014). Unlike static paper-and-pencil training or exercise alone, motion-based video games require individuals with MCI to maintain postural control while processing dynamic visual stimuli, thereby engaging sensorimotor cortices and prefrontal regulatory circuits. Corresponding improvements in MMSE and MoCA in our results may thus reflect a dual enhancement of cerebrovascular function and synaptic plasticity, supporting a physiological foundation for slowing cognitive decline.

The intrinsic enjoyment and immediate feedback embedded in video games play a crucial affective role in cognitive gains among individuals with MCI. Apathy, anxiety, and depressive symptoms are common in MCI and can further suppress cognitive performance. Neuroimaging work by Kühn et al. (2011) demonstrated that the sense of achievement during gameplay triggers dopaminergic release in the striatum (Kühn et al., 2011). Dopamine not only modulates mood but also acts as a gating mechanism for hippocampal long-term potentiation (LTP), facilitating memory consolidation and learning efficiency. Compared with monotonous traditional rehabilitation, the high adherence and intrinsic motivation associated with video gaming promote active engagement, and this positive emotional state can enhance cognitive test performance while potentially mitigating cortisol-related hippocampal vulnerability.

## 5 Limitations

Although this systematic review and meta-analysis was conducted in accordance with PRISMA guidelines, several methodological limitations should be acknowledged. First, the number of included studies and their cumulative sample size were relatively modest. The limited evidence base reduces the statistical power of the analyses and may increase susceptibility to publication bias, thereby inflating the estimated effects of video game interventions. Second, marked heterogeneity was observed across studies. Despite the application of subgroup analyses, the heterogeneity could not be fully accounted for, which constrains the ability to propose a uniform and evidence-based intervention protocol. Third, the nature of video game interventions presents inherent challenges to blinding. Double blinding of participants and intervention providers is largely unfeasible. Although blinding of outcome assessors may have been implemented, participants' knowledge of their allocation could contribute to expectancy-driven improvements, introducing a potential Hawthorne effect and increasing the risk of performance bias. Fourth, the scarcity of long-term follow-up data limits conclusions regarding the durability of intervention effects. Most studies employed relatively short intervention durations, typically between 4 and 12 weeks, and few reported cognitive outcomes after the cessation of training. Consequently, the persistence of cognitive benefits and the potential of video game–based interventions to delay progression from MCI to Alzheimer's disease remain uncertain. Future trials should incorporate extended follow-up periods and longitudinal designs to better characterize long-term efficacy.

## 6 Conclusion

This systematic review and meta-analysis indicates that video game-based interventions, particularly those integrating physical activity (exergames), are a promising non-pharmacological strategy for enhancing global cognition and executive functioning in older adults with mild cognitive impairment. The observed improvements in MoCA, MMSE, and TMT scores suggest that such interventions may offer clinically meaningful benefits. However, given the limited number of studies and high heterogeneity, future research should prioritize standardized protocols and larger-scale randomized controlled trials with long-term follow-up to validate these findings and elucidate the optimal intervention parameters.

## Data Availability

The original contributions presented in the study are included in the article/Supplementary material, further inquiries can be directed to the corresponding author.
