# Supplementary material for: The effects of video games on cognitive function in older adults with mild cognitive impairment: a meta-analysis
Source: Front Aging Neurosci. 2026 Jan 23;17:1756970. doi: 10.3389/fnagi.2025.1756970 (PMC12876208; doi:10.3389/fnagi.2025.1756970)
Supplement: Supplementary file 1 [file Table_1.docx]

**Supplementary S-Table 1: Search strategy for PubMed as October 12, 2025.**

| **Search** | **Query** | **Results** |
| --- | --- | --- |
| #1 | Mild Cognitive Impairment[MeSH Terms] | 50,511 |
| #2 | Mild Cognitive Impairment[Title/Abstract] OR Cognitive Dysfunction[Title/Abstract] OR Cognitive Impairments[Title/Abstract] OR MCI[Title/Abstract] | 78,022 |
| #3 | #1 OR #2 | 104,223 |
| #4 | Video Games[MeSH Terms] | 8,717 |
| #5 | Video Games[Title/Abstract] OR Games, Video[Title/Abstract] OR Game, Video[Title/Abstract] OR Video Game[Title/Abstract] OR Computer Games[Title/Abstract] OR Computer Game[Title/Abstract] OR Game, Computer[Title/Abstract] OR Games, Computer[Title/Abstract] | 7,259 |
| #6 | #4 OR #5 | 12,607 |
| #7 | Review[Title/Abstract] OR Overview[Title/Abstract] OR Meta[Title/Abstract] | 2,888,996 |
| #8 | #3 AND #6 NOT #7 | 156 |

**Supplementary S-Table 2: Search strategy for Web of Science as at October 12, 2025**

| **Search** | **Query** | **Results** |
| --- | --- | --- |
| #1 | TS=(Video Games OR Games, Video OR Game, Video OR Video Game OR Computer Games OR Computer Game OR Game, Computer OR Games, Computer ) | 24481 |
| #2 | TS=(Mild Cognitive Impairment OR Cognitive Dysfunction OR Cognitive Impairments OR MCI) | 213276 |
| #3 | TS=(Review OR Overview OR Meta) | 3022606 |
| #4 | #1 AND #2 | 402 |
| #5 | **#4 NOT #3** | 302 |

**Supplementary S-Table 3: Search strategy for EMBASE as at October 12, 2025**

| Search | Query | **Results** |
| --- | --- | --- |
| #1 | mild cognitive impairment'/exp | 50666 |
| #2 | mild cognitive impairment':ab,ti OR 'cognitive dysfunction':ab,ti OR 'cognitive impairments':ab,ti OR 'mci':ab,ti | 121060 |
| #3 | 'video game'/exp OR 'video game' | 11574 |
| #4 | 'video games':ab,ti OR 'games, video':ab,ti OR 'game, video':ab,ti OR 'video game':ab,ti OR 'computer games':ab,ti OR 'computer game':ab,ti OR 'game, computer':ab,ti OR 'games, computer':ab,ti | 9279 |
| #5 | #1 OR #2 | 132617 |
| #6 | #3 OR #4 | 14255 |
| #7 | 'review':ti OR 'overview':ti OR 'meta':ti | 1213333 |
| #8 | #5 AND #6 | 294 |
| #9 | #8 NOT #7 | 252 |

**Supplementary S-Table 4:Search strategy for Cochrane as at October 12, 2025**

| Search | Query | **Results** |
| --- | --- | --- |
| #1 | MeSH descriptor: [Cognitive Dysfunction] explode all trees | 4807 |
| #2 | (Mild Cognitive Impairment):ab,ti,kw OR (Cognitive Dysfunction):ab,ti,kw OR (Cognitive Impairments):ab,ti,kw OR (MCI):ab,ti,kw | 19680 |
| #3 | MeSH descriptor: [Video Games] explode all trees | 1367 |
| #4 | (Video Games):ab,ti,kw OR (Games, Video):ab,ti,kw OR (Game, Video):ab,ti,kw OR (Video Game):ab,ti,kw OR (Computer Games):ab,ti,kw OR (Computer Game):ab,ti,kw OR (Game, Computer):ab,ti,kw OR (Games, Computer):ab,ti,kw | 4295 |
| #5 | #1 OR #2 | 19715 |
| #6 | #3 OR #4 | 4348 |
| #7 | (Review)ti OR (Overview)ti OR (Meta-analysis)ti | 10879 |
| #8 | #5 AND #6 | 299 |
| #9 | #8 NOT #7 in Trials | 292 |

**Supplementary S-Table 5:Search strategy for CINAHL as at October 12, 2025**

| Search | Query | Results |
| --- | --- | --- |
| #1 | Video Games OR Video Game) AND (Mild Cognitive Impairment OR MCI) NOT (Review OR Overview OR Meta) | 26 |

**Supplementary S-Table 6:Search strategy for Mediline as at October 12, 2025**

| Search | Query | Results |
| --- | --- | --- |
| #1 | ((Video Games OR Video Game) AND (Mild Cognitive Impairment OR MCI)) NOT ((Review OR Overview OR Meta)) | 88 |

**Supplementary S-Table 7: Detailed descriptions of video game interventions used in included studies**

| Study (Author, Year) | Gaming Platform / Device | Game Name / Software | Detailed Gameplay & Scenarios |
| --- | --- | --- | --- |
| Saeed et al. (2024) | Wobble-board Exergame (Custom-made with Unity 3D) | The Maze Game | Participants stand on a custom wobble board connected to a screen. By shifting their center of gravity (leaning forward, backward, left, or right), they control a virtual ball to navigate through a maze. The goal is to maneuver the ball into a target hole while avoiding false holes. The game involves visual-spatial planning and continuous postural adjustments. |
| Savulich et al. (2017) | iPad Tablet (Touchscreen) | Game Show (Wizard Memory Game) | A gamified cognitive training app based on episodic memory. The player acts as a contestant on a "Game Show" and competes against a virtual opponent. The task requires remembering the specific locations of geometric patterns associated with different background themes to win gold coins. The difficulty (number of patterns) is adaptive based on performance. |
| Arshad et al. (2023) | Xbox 360 Kinect (Motion sensor) | Kinect Adventures | A full-body motion-sensing game suite. Participants engage in five specific mini-games: 1. 20,000 Leaks: Players use hands and feet to plug leaks in a glass cube underwater. 2. River Rush: Players jump and move laterally to steer a raft. 3. Rally Ball: Players hit balls at targets using their limbs. 4. Reflex Ridge: A track-and-field style obstacle course requiring jumping and ducking. 5. Space Pop: Players fly in zero-gravity to pop bubbles. |
| Liu et al. (2022) | Kinect Sensor + PC | Long-Good Tai Chi Training System | An interactive Augmented Reality (AR) Tai Chi training system. A virtual instructor demonstrates 6 classic Yang-style Tai Chi forms (e.g., "Parting the Wild Horse's Mane", "Cloud Hands") on screen. The Kinect sensor captures the user's real-time skeleton, and the system provides immediate visual feedback (green/red lights) to correct posture and accuracy. |
| Amjad et al. (2019) | Xbox 360 Kinect (Motion sensor) | Kinect Adventures | Participants performed the same five mini-games as in Arshad et al. (2023): 20,000 Leaks, River Rush, Rally Ball, Reflex Ridge, and Space Pop. The intervention required gross motor movements (upper and lower limbs) combined with cognitive demands such as rapid reaction, inhibition, and spatial attention. |

**Table 8. Subgroup analysis of the effects of video games on cognitive function.**

| Outcome | Subgroup | Categories | No. of Studies | Effect Size (95% CI) | Heterogeneity (P) |
| --- | --- | --- | --- | --- | --- |
| MoCA (MD) | Control Type | Active Control | 3 | 3.50 [3.26, 3.73] | < 0.001 |
|  |  | Passive Control | 1 | 1.80 [1.54, 2.06] |  |
|  | Duration | Short (≤ 6 weeks) | 2 | 3.49 [3.25, 3.72] | < 0.001 |
|  |  | Long (> 6 weeks) | 2 | 1.86 [1.61, 2.12] |  |
|  | Frequency | High (> 3x/week) | 2 | 3.49 [3.25, 3.72] | < 0.001 |
|  |  | Low (≤ 3x/week) | 2 | 1.86 [1.61, 2.12] |  |
| MMSE (MD) | Control Type | Active Control | 2 | 2.40 [2.08, 2.72] | 0.086 |
|  |  | Passive Control | 1 | 1.30 [0.09, 2.51] |  |
|  | Game Type | Exergame (Motion) | 2 | 2.40 [2.08, 2.72] | 0.086 |
|  |  | iPad (Static) | 1 | 1.30 [0.09, 2.51] |  |
| TMT-A (SMD) | Control Type | Active Control | 3 | -1.22 [-1.56, -0.87] | < 0.001 |
|  |  | Passive Control | 1 | -7.45 [-9.37, -5.52] |  |
|  | Frequency | High (> 3x/week) | 2 | -1.56 [-2.05, -1.08] | 0.39 |
|  |  | Low (≤ 3x/week) | 2 | -1.26 [-1.75, -0.78] |  |
| TMT-B (SMD) | Duration | Short (≤ 6 weeks) | 2 | -2.05 [-2.61, -1.48] | < 0.001 |
|  |  | Long (> 6 weeks) | 2 | -0.43 [-0.89, 0.04] |  |

Note: MD = Mean Difference; SMD = Standardized Mean Difference. P indicates the significance of the difference between subgroups.
